# War-related traumatic brain injuries during the Syrian armed conflict in Damascus 2014–2017: a cohort study and a literature review

**DOI:** 10.1186/s12873-023-00799-6

**Published:** 2023-03-29

**Authors:** Ibrahem Hanafi, Eskander Munder, Sulafa Ahmad, Iman Arabhamo, Suzan Alziab, Noor Badin, Ahmad Omarain, Mhd Khaled Jawish, Muhannad Saleh, Vera Nickl, Tamara Wipplinger, Christoph Wipplinger, Robert Nickl

**Affiliations:** 1 Division of Neurology, Department of Internal Medicine, Faculty of Medicine, Damascus University, Mazzah, Damascus, Syria; 2 Faculty of Medicine, Syrian Private University, Mazzah, Damascus, Syria; 3 Faculty of Medicine, Damascus University, Mazzah, Damascus, Syria; 4 Division of Neurosurgery, Department of Surgery, Damascus Hospital, Damascus, Syria; 5 Department of Neurosurgery, Würzburg University, Würzburg, Germany; 6 Department of Biobehavioral Sciences, Teachers College, Columbia University, New York, USA

**Keywords:** Explosion injury, Gunshot injury, Neurological injury, Syrian armed conflict, War-related injury, Combat injury, Brain injury

## Abstract

**Background:**

The decade-long Syrian armed conflict killed or injured more than 11% of the Syrian population. Head and neck injuries are the most frequent cause of war-related trauma, about half of which are brain injuries. Reports about Syrian brain trauma victims were published from neighboring countries; However, none are available from Syrian hospitals. This study aims to report war-related traumatic brain injuries from the Syrian capital.

**Methods:**

We conducted a retrospective cohort study between 2014 and 2017 at Damascus Hospital, the largest public hospital in Damascus, Syria. Target patients were the victims of combat-related traumatic brain injuries who arrived alive and were admitted to the neurosurgery department or to another department but followed by the neurosurgery team. The collected data included the mechanism, type, and site of injury based on imaging findings; types of invasive interventions; intensive-care unit (ICU) admissions; as well as neurological status at admission and discharge including several severity scales.

**Results:**

Our sample consisted of 195 patients; Ninety-six of them were male young adults, in addition to 40 females and 61 children. Injuries were caused by shrapnel in 127 (65%) cases, and by gunshots in the rest, and most of them (91%) were penetrating. Sixty-eight patients (35%) were admitted to the ICU, and 56 (29%) underwent surgery. Neurological impairment was reported in 49 patients (25%) at discharge, and the mortality rate during hospitalization was 33%. Mortality and neurological impairment associated significantly with higher values on clinical and imaging severity scores.

**Conclusions:**

This study captured the full spectrum of war-related brain injuries of civilians and armed personnel in Syria without the delay required to transport patients to neighboring countries. Although the clinical presentation of injuries at admission was not as severe as that in previous reports, the inadequate resources (i.e., ventilators and operation rooms) and the lack of previous experience with similar injuries might have resulted in the higher mortality rate. Clinical and imaging severity scales can provide a handy tool in identifying cases with low probability of survival especially with the shortage of personal and physical resources.

**Supplementary Information:**

The online version contains supplementary material available at 10.1186/s12873-023-00799-6.

## Introduction

The decade-long Syrian armed conflict (SAC) has become the most devastating humanitarian crisis of our time, affecting the lives of millions, and keeping Syria the most dangerous country worldwide for five years [1, 2]. Various lethal weapons have been used against both military personnel and civilians, causing death to nearly 500,000 and injuries to 2,000,000 citizens, which is approximately 11.5% of the Syrian population [3–5]. The methods of attack included air strikes, artillery shelling, missiles, shrapnel, gunshots, and others [6].

Historically, most reports about armed conflicts were focused on injuries in military staff. Head and neck injuries made up 15–30% of the injuries of the British forces since 1900 [7], and 42% of all deadly penetrating injuries for British soldiers in Afghanistan affected this area [8]. This demonstrates the essential role of neurosurgery, the war-born surgical specialty, in combat-related injuries, which increased due to the rapid development of weaponry and the limited resources in war zones [9]. Nevertheless, little consideration from published reports was oriented towards civilian victims [10].

During the SAC, more than 70% of war-related injuries occurred to civilians [11], and the collapsing healthcare system became overwhelmed with casualties soon after the spread of conflicts because of several synergic reasons. Firstly, there was a lack of experience in transporting, triaging, assessing, and managing war-related injuries in a previously peaceful country. Secondly, Syria faced a dramatic reduction in numbers of healthcare professionals [12–14], a shortage in emergency medical equipment (i.e., ventilators, surgical equipment, and antiseptics) and medications (i.e., antibiotics and sedatives) [15, 16], a collapsing economy, and an early destruction of pharmaceutical companies [17, 18]. Furthermore, these obstacles were amplified by the rapid deterioration of local health facilities, especially after half of the country’s hospitals ran out of service and 90% of its ambulances were damaged or stolen [18].

Several reports on war-related injuries in the SAC have been published in neighboring countries [19–23], and a systematic review found that injuries to the head, face, and neck were the most common (37%) [24]. Brain damage and hemorrhage were also the most common causes of death in Syrians who presented to Turkish and Jordanian trauma centers [25, 26]. However, these reports presented only the number of patients who managed to cross the borders to neighboring countries and suggested that a study reporting the cases treated in a local Syrian medical facility could provide a more accurate estimate of injury patterns. Therefore, we aimed to report a sample of war-related brain injuries in the Syrian capital between 2015 and 2017.

### Patients and methods

#### Study design

We conducted a retrospective cohort study at Damascus Hospital, the largest public hospital in the Syrian capital that provides free medical services for civilians and armed personnel in central and rural Damascus.

#### Inclusion and exclusion criteria

The paper records of all patients admitted to the neurosurgery ward in the period between December 2014 and November 2017 were retrospectively screened. Patients with new brain injuries due to explosions, gunshots or stabbing were included in this analysis. Patients who suffered from brain injuries but were admitted to other departments due to more life-threatening injuries were also considered. No age or gender restrictions were applied in this study. However, victims who arrived dead or died in the emergency department before completing the admission procedures to the hospital could not be identified nor included for this study. Patients with other non-combat related aetiologies like falls, car accidents, and tumours were excluded, as well as follow-up patients and patients with pure peripheral or spinal cord injuries.

#### Data collection

Data were collected and entered to a form created on Microsoft Office Access Professional Plus 2013 by two collaborators. The collected data included demographic characteristics and the mechanism, type, and site of injury based on brain computed tomography (CT) findings as well as surgeries, intensive-care unit (ICU) admission, and neurological status at discharge. Additionally, we assessed Glasgow Coma Scale (GCS) score [27], the modified version of the Head Injury Severity Scale (HISS) [28], The Revised Trauma Score (RTS) [29], The 2008 update of the Abbreviated Injury Scale 2005 (AIS), the Injury Severity Scale (ISS) [30, 31], and the probability of survival (PoS) by means of the Trauma and Injury Severity Score (TRISS) [32].

#### Data analysis

Data were exported into the Statistical Package for the Social Sciences version 23.0 (SPSS Inc., Chicago, IL, United States) for analysis. Chi-square and Fisher’s exact tests were used to investigate the association between categorical variables, while independent samples Mann Whitney U test, Receiver Operating Characteristics (ROC) Curve, and area under the curve (AUC) were used to study continuous variables. An alpha value of 0.05 was used to determine the threshold of statistical significance.

## Results

### Population demographics

During the targeted period, a total of 3911 patients were admitted to the neurosurgery ward or visited by the staff of neurosurgery department in other wards at Damascus Hospital. The number of quarterly admissions ranged between 275 and 379, with the highest record between March and May 2015 (Supplementary Fig. 1). Two-hundred fifty-five patients (6.5% of the total admissions) had new war-related injuries to the central nervous system (CNS), while other admissions happened for war-independent traumatic injuries, peripheral traumatic neurological injuries, as well as typical elective and emergent neurosurgeries. For the war related injuries (255 patients), the highest frequency of admissions was recorded between September and November 2015 (Supplementary Fig. 2), and out of them, 195 patients (76.5%) had at least one injury to the brain and therefore matched our inclusion criteria. Patients’ age ranged between one and 63 years with a median of 25, and 96 of the victims (49.2%) were male young adults (Table 1).

**Table 1:** Population characteristics of victims with war-related traumatic brain injuries

Characteristics	Categories	Number	Percentage
Age groups	Children	61	31.3
	Young adults^a^	111	56.9
	Old adults^a^	23	11.8
Sex	Males	154	79.0
	Females	41	21.0
Mechanism of Injury	Shrapnel	127	65.1
	Gunshot	63	32.3
	Stabbing	5	2.6

^a^Young adults are between 18–44 years old, while old adults are 45 years old or older

### Types and sites of injuries

All recruited patients underwent CT scans for the head and neck, and 11 of them (5.6%) had scans for the spine as well. A total of 178 patients (91.3%) had penetrating injuries, and 43 patients (22.1%) presented with extracranial herniation of brain tissue. The most common types of injury were metal fragments (*n* = 124; 63.6%) and intracerebral haemorrhage (*n* = 67; 34.4%). Injuries were equally distributed between the right and left hemispheres (57.4% and 54.9% respectively) and crossed the midline in 36 patients (18.5%). Accompanying spinal cord injuries were found in eight patients, while other accompanying injuries mainly included skull fractures. Forty-eight patients (24.6%) had injuries to more than one body area, and more than 70% of brain injuries were considered serious or severe (AIS > 2) (Table 2).

**Table 2:** Types and sites of war-related traumatic brain injuries

Injury	Number	Percentage	Injury	Number	Percentage
Penetrating injury	178	91.3	Injured hemisphere^a^		
Type of injury			Right lobes	112	57.4
Metal foreign body	124	63.6	Left lobes	107	54.9
Intracerebral hemorrhage	67	34.4	Crossing midline	36	18.5
Loss of brain material	43	22.1	Other injuries		
Subarachnoid hemorrhage	32	16.4	Vault skull fracture	156	80
Bony foreign body	29	14.9	Basilar skull fracture	19	9.7
Subdural hemorrhage	27	13.8	CSF leakage	15	7.7
Cerebral contusion	23	11.8	Cranial nerves	12	6.2
Intraventricular hemorrhage	16	8.2	Pneumocephalus	10	5.1
Epidural hemorrhage	2	1.0	Vertebral fracture	6	3.1
Other injuries	10	5.1	Count of body parts injured^b^		
Site of Injury^a^			One	147	75.4
Left parietal lobe	56	28.7	Two	36	18.5
Right parietal lobe	48	24.6	Three	9	4.6
Right frontal lobe	48	24.6	Four	3	1.5
Left frontal lobe	42	21.5	The most severe head injury^b^		
Right temporal lobe	41	21.0	Minor (1)	2	1.0
Right occipital lobe	31	15.9	Moderate (2)	16	8.2
Left temporal lobe	27	13.8	Serious (3)	70	35.9
Left occipital lobe	25	12.8	Severe (4)	67	34.4
Brainstem	3	1.5	Critical (5)	39	20.0
			Incompatible with life (6)	1	0.5

^a^The site of injury was lost from the records of 12 patients

^b^Categorized according to the Abbreviated Injury Score (AIS)

#### Cases management

Twenty-nine (14.9%) patients underwent immediate surgery within less than 24 h after admission, while 27 (13.8%) patients underwent delayed surgery. Immediate surgery rates were similar throughout the GCS severity levels at admission. However, less delayed surgeries were performed in the severe GCS group (*n* = 4; 6.9%) in comparison to the moderate (*n* = 5; 14.7%) and mild groups (*n* = 18; 35.3%). The most common surgeries were foreign body removal and repairing meninges, followed by hemorrhage evacuation. The length of stay at the hospital ranged from one to 115 days with a median of five days. About a third of the patients (68 patients) were admitted to the ICU, and the median length of stay there was 4.5 days with a range of one to 113 days. Fifty-four of the patients admitted to the ICU (79.4%) died during hospitalization (52 of them during their stay in the ICU), while 11 patients (8.7%) died during hospitalization but without being admitted to the ICU﻿ (Table 3).

**Table 3:** Management plans and ICU admissions

Management	Category	Number	Percentage
Management	Conservative Medical Care	139	71.3
	Delayed Surgery	27	13.8
	Immediate Surgery	29	14.9
Surgical Procedure	Foreign Body Removal	21	10.8
	Repair of meninges	20	10.3
	Hemorrhage drainage	6	3.1
	Abscess Drainage	1	0.5
	Shunt Insertion	1	0.5
	Other	7	3.6
Length of stay		5^b^	3-10^c^
ICU admission		68	34.9
Length of stay in ICU (*n* = 68)^a^		4.5^b^	2-10^c^
ICU Discharge (*n *= 68)^a^	Death	52	76.5
	To the neurosurgery ward	16	23.5

^a^After exclusion of patients who were not admitted to the ICU

^b^Median

^c^Interquartile range

On the other hand, 13 patients of those who underwent surgery (23.2%) died compared to 52 (37.4%) of those managed conservatively. After excluding patients who had a GCS of three at admission, patients with severe GCS who underwent surgery had a lower mortality rate (*n* = 8, 61.5%) than those who did not undergo surgery (*n* = 33, 86.8%; *P* = 0.047). In contrast, the moderate GCS group had a significantly higher rate of discharge deficit for those who underwent surgery (*n* = 6, 75.0%) in comparison with their counterparts who did not (*n* = 4, 28.6%; *P* = 0.035; Table 4).

**Table 4:** Mortality during admission and neurological deficits at discharge among surgery and GCS groups

GCS^a^	Surgery	Total number	Mortality during admission [n (%)]	*P* value^c^	Discharge deficit [n (%)]	*P* value^c^
Severe^b^	Yes	13	8 (61.5)	0.047	4 (80.0)	0.292
	No	38	33 (86.8)		5 (100.0)	
Moderate	Yes	11	3 (27.3)	0.498	6 (75.0)	0.035
	No	23	9 (39.1)		4 (28.6)	
Mild	Yes	32	2 (6.3)	0.658	13 (43.3)	0.070
	No	71	3 (4.2)		17 (25.0)	
Total	Yes	56	13 (23.2)	0.140	23 (53.5)	0.009
	No	132	45 (34.1)		26 (29.9)	

^a^ GCS: Glasgow coma score at admission

^b^ The seven patients who scored three on the GCS were excluded from the whole analysis because none of them underwent surgery (and all of them died), leaving only 188 patients for the analysis of this table

^c^ Chi square test

#### Status at discharge

Thirty-nine patients (69.6%) underwent surgery without complications, and 17 (30.4%) had at least one complication. The most common complications were infectious including abscess formation and meningitis, followed by seizures and surgical site infections. Additionally, ten patients (7.2%) in the conservatively managed group had complications that also varied between meningitis, herniation, hematoma, and seizures. Overall, 65 patients (33.3%) died during hospitalization and 49 (25.1%) had neurological deficits at discharge. The most frequent discharge deficits were paresis, paralysis, and aphasia (Table 5). Twenty-three of surgically managed patients (53.5%) had neurological deficits at discharge in comparison to 26 (29.9%) of those conservatively managed (P = 0.009). More specifically, 15 out of 21 immediate surgeries were associated with neurological deficits compared to eight out of 22 among delayed surgeries.

**Table 5:** Surgical complications, progression of conservatively managed cases and status at discharge

**Outcomes**		**Number**	**Percentage**
Type of surgical complication (*n* = 56)	Abscess	8	14.3
	Meningitis	8	14.3
	Seizures	5	8.9
	Surgical site infection	3	5.4
	Fistula	2	3.6
	Hydrocephalus	1	1.8
	Hematoma	1	1.8
	Not in the CNS	1	1.8
Progression in conservative cases (*n* = 139)	Meningitis	3	2.2
	Herniation	2	1.4
	Hematoma	1	0.7
	Seizure(s)	1	0.7
	Not in the CNS	3	2.2
Status at Discharge	Healthy	81	41.5
	Discharge deficit	49	25.1
	Death	65	33.3
GCS at Discharge (*n* = 130)	Mild	126	96.9
	Moderate	3	2.3
	Severe	1	0.8
Discharge deficits (*n* = 130)	Paresis	29	22.3
	Paralysis	9	6.9
	Aphasia	7	5.4
	Incontinence	3	1.5
	Blindness	3	2.3
	Numbness	1	2.3
	Coma	1	0.8
	Others	7	3.6

^a^ GCS: Glasgow coma score at discharge categorized into mild (13-15), moderate (9-12) and severe (3-8) injury groups

#### Severity scales at presentation and status at discharge

Fifty-eight patients had severe GCS (29.7%) with a mortality rate of 82.8% and nine of the ten surviving patients in this group showed neurological deficits at discharge. In contrast, only five of the 103 patients with mild GCS (4.9%) died, and 73 patients (70.9%) were discharged free of deficits. The GCS score upon admission correlated significantly with mortality and neurological deficits at discharge (*P* ≤ 0.001).

Comparable findings also applied to HISS, where the mortality rate was 82.8% in severe, 36.1% in moderate, 8% in mild, and 2.6% in minimal injuries (*P* < 0.001). Neurological deficits also happened in 90%, 43.5%, 30.4% and 31.1%, respectively (*P* = 0.003). Similarly, the higher RTS scores significantly associated with mortality (*P* < 0.001) but did not reach statistical significance in its association with neurological deficits (*P* = 0.365). Additionally, patients with critical ISS had a mortality rate of 70.4%, compared to 4.4% in the minor/moderate ISS group (*P* < 0.001). Deficits at discharge in the former group were also 28.5% higher than in the latter (*P* = 0.028).

The predicted PoS among patients in this study had a median of 97.6% (interquartile range (IQR): 77.2–99.1%). It was 66.0% (42.2–89.0%) for patients who died during hospitalization and 98.8% (97.4–99.1%) among survivals (*P* < 0.001). A ROC Curve of the PoS and the survival status had an AUC of 0.926 (*P* < 0.001; 95% confidence interval: 0.889-0.962), representing an excellent predictor. By setting 97.5% as a cutoff, sixty-four patients (66.0%) of those with lower PoS died, compared to only one patient (1.0%) of those with higher PoS (*P* < 0.001). Additionally, the survivals from the former group had more than twice the rate of neurological deficits in the latter group at discharge (*P* < 0.001; Table 6).

**Table 6:** Clinical assessment scores at presentation and their association with mortality and neurological deficits at discharge

Score	Total			Mortality			Deficit at discharge (*n* = 130)		
	Severity	Number	Percentage	Number	Percentage	*P* value	Number	Percentage	*P* value
GCS (Glasgow Coma Score) at admission	Severe	58	29.7	48	82.8	< 0.001^ab^	9	90.0	0.001^ab^
	Moderate	34	17.4	12	35.3		10	45.5	
	Mild	103	52.8	5	4.9		30	30.6	
Head injury severity scale (HISS)	Severe	58	29.7	48	82.8	< 0.001^ab^	9	90.0	0.003^ab^
	Moderate	36	18.5	13	36.1		10	43.5	
	Mild	25	12.8	2	8.0		7	30.4	
	Minimal	76	39.0	2	2.6		23	31.1	
Revised trauma score (RTS)	High severity	29	14.9	24	82.8	< 0.001^bc^	3	60.0	0.365^c^
	Low severity	166	85.1	41	24.7		46	36.8	
Injury severity scale (ISS)	Critical	54	27.7	38	70.4	< 0.001^ab^	9	56.3	0.028^ab^
	Severe	66	33.8	24	36.4		20	47.6	
	Moderate/Minor	75	38.5	3	4.4		20	27.8	
Probability of survival (PoS)	< 97.5%	97	49.7	64	66.0	< 0.001^bc^	22	66.7	< 0.001^bc^
	≥ 97.5%	98	50.3	1	1.0		27	27.8	

^a^Chi square test

^b^ Statistically significant at the level of 0.05

^c^ Fisher exact test

## Discussion

This study was the first to investigate war-related traumatic brain injuries inside Syria during the SAC. It was conducted in the largest public hospital in the Syrian capital presenting various brain injuries caused by different mechanisms and resulting in distinct severity levels. To the best of our knowledge, only four studies reported combat related TBI from the SAC, and all of them were of a smaller size, lasted for a shorter period, had less representation of females and children, and were published from neighbouring countries (Table 7). This maximizes the importance of this study as it reflects a more representative picture of the civilian victims including the most vulnerable groups. It also minimized the unavoidable reporting bias in similar studies on Syrian refugees in neighbouring countries by excluding the delay from the instant of the injury to presentation and the challenge of crossing the borders and reaching health care facilities in these countries [21].

**Table 7:** A comparison of published articles on war-related traumatic brain injuries during the Syrian armed conflict

Study—Country	Study period	Patients’ count	Male/ Female^a^	Shrapnel/ Gunshot^a^	GCS^ab^	Surgery rate^a^	Mortality rate^a^
Aras et al. 2014—Turkey [23]	04.2011–01.2013	186	166/20 (89.2) Including 16 children (8.6)	126/60 (67.7)	≤ 7: 102 (54.8) ≥ 8: 84 (45.1)	88 (47.3)	59 (31.7)
Barhoum et al. 2015—Israel [21]	03.2013–05.2014	66	54/5 (91.5) + 7 children (10.6)^c^	25/10 (71.4)^d^	Average (range): 9 (3–15)	46 (69.6)	3 (4.5)
Can et al. 2017—Turkey [20]	01.2014–06.2014	104	96/8 (92.3)	All gunshot	≤ 6: 17 (16.3) ≥ 7: 87 (83.7)	-	38 (36.5)
Jamous et al. 2019—Jordan [22]	06.2012–11.2013	44	38/6 (86.4)	33/11 (75.0)	≤ 7: 20 (45.4) 8–12: 6 (13.6) ≥ 13: 18 (41.0)	25 (56.8)	11 (25.0)^e^
Our study	12.2014–11.2017	195	154/41 (79.0) including 61 children (31.3)	127/68 (65.1)	≤ 8: 58 (29.7) 9–12: 34 (17.4) ≥ 13: 103 (52.8)	56 (25.7)	65 (33.3)

^a^The data are presented as N (%)

^b^ GCS: Glasgow coma score at admission

^c^The seven children were not classified into males and females

^d^The injuries included 31 (47.0%) patients who had assault or combat-independent injuries, but we could not exclude them from the presented values

^e^ In contrast to all other studies, this mortality rate is calculated during the whole follow-up period that ranged from one to 15 months

Young adult males were the most prone group to suffer from injury in the sample of this study. This might be justified by the local traditional custom in which young males are responsible for families’ income and therefore work outdoors more commonly [33]. Meanwhile, elderly people, women, and children are more likely to stay shielded in houses, schools, or other closed spaces [34]. However, our present study had more females and children compared to other reports on the SAC [21, 22, 25]. This might be explained by the fact that Damascus, unlike other governorates, did not endure any open field battles, and many of the attacks targeted residential neighborhoods, schools and markets [33]. Overall, the similarities between our sample and other studies published from Syrian hospitals [33, 35, 36], and the minimization of presentation delay, selectivity, and language barriers in centers abroad [37, 38], may have made this study more representative of the full spectrum of war-related brain injuries during the SAC.

The findings of this study were in line with comparable studies about abdominal injuries published from hospitals in Damascus, as it found explosions to be the most frequent mechanism of injury followed by gunshot [33, 35]. In contrast, published articles from neighboring countries agreed that gunshot is the most prevalent cause [24, 27, 39, 40], which might be possibly attributed to the high severity of multiorgan shrapnel injuries that increased the challenge of crossing the borders and presenting to these centres. This might be also due to the differences between conflict areas as the patients of these studies came from areas near the borders where fire exchange and open battles were most likely to happen. These findings do not apply to studies from the Syrian cities where missiles and mortars were the most commonly used weaponry [6].

Considering the type of injury, acute subdural hematoma was more common in this study than the study of Barhoum and colleagues [21], while subarachnoid haemorrhage and epidural hematoma were less commonly encountered. This may be explained by the fact that acute subdural hematoma is associated with more severe head injuries in comparison to the latter two, which might have been underreported in our patients’ records [41–44]. The neuroimaging findings were also collected from the reports attached to the medical records, because the original images were not available anymore. Therefore, we could not control for the lack of documentation of less severe injuries, an inevitable consequence of the huge workload caused by the waves of injured victims presenting to our center after explosions and armed clashes. In contrast, patients in the study of Barhoum and colleagues were first admitted to a field hospital, stabilized, and then transferred to the medical center where the study took place. This could have given the staff enough time to improve the level of documentation and also the preparedness for probable surgical management [21]. However, one can also argue that our study is more accurate in its representation since we only included patients who presented and were triaged, managed, and followed at the same center by the same neurosurgery team.

Surgical decision making has been a debatable issue in war-related neurosurgical injuries. This is especially true when patients present conscious and in a relatively good condition [21]. Less surgeries were performed in this study than in other comparable studies (Table 7) [21–23]. This could be attributed to multiple factors including the predominance of mild GCS reduction in the sample (Table 7), the high probability of complications after surgical interventions (Table 5) [45], and the extremely limited availability of ventilators and ICU beds [46]. Therefore, the neurosurgery team tended to be highly conservative, and only performed surgeries for patients with promising outcomes in order to reduce the length of stay in ICUs and the consumption of the already limited resources. These tendencies are also supported with the fact that although the applied surgeries were associated with a higher survival in the severe GCS group, it was also associated with a higher occurrence of neurological deficits at discharge in the moderate GCS group (Table 4). Unfortunately, long term outcomes such as Glasgow outcome scale could not be evaluated due to the retrospective design and the highly dynamic population during this period of political insecurity. A comparison of the mechanisms of care during the time of the study to the period before the war was also not possible, due to the variety of contributing factors that include the loss of experienced personnel [12, 14], the sharp shortage of equipment and supplies, as well as the multiplied flow of patients after half of Damascus hospitals were destroyed [47].

Infectious complications after combat trauma are common and challenging [48], and they were the most common complication in the sample of this study. This can be explained by the high antibiotic resistance in the Syrian community, which existed even before the start of the war because of the loose regulations and the widespread practices of self-medication [49]. The poor sterilization of overloaded operation rooms, as well as the loose hygienic sanitary measures also aggravated the probabilities of such complications during the SAC [19]. Although the mortality rate in our analysis (33.3%) excluded patients who arrived dead or died before admission, it was still significantly higher than in similar studies in Jordanian and Israeli centres [21, 22]. Meanwhile, although it was comparable to the mortality rate in the two Turkish studies, it is worth mentioning that one of them had many more patients in the severe GCS category [23], and the other recruited only gunshot injuries [20], also suggesting a worse outcome in our sample (Table 7). A possible reason for that might be the short distances and rapid transportation of victims in Damascus. This might have captured critical patients that would not have had enough resilience to cross the borders and reach medical centers abroad alive. It might also be explained by the shortage of staff, equipment, and preparedness in the medical centres inside Syria. Therefore, we believe that this article provides a full image of the presentation, management, and outcome of war-related traumatic brain injuries in the largest hospital treating civilians and combatants during three years of the SAC in Damascus.

## Conclusions

War-related brain injuries during the Syrian armed conflict presented a huge challenge for the Syrian healthcare system. Although several reports presented such injuries from neighboring countries, Syrian health facilities faced a higher challenge to manage these cases. Patients’ status at presentation to our center was less severe than in other reports, however, the inadequate resources (i.e., ventilators and operation rooms) as well as the lack of previous experience with war-related brain injuries resulted in a high mortality and complications rate among victims.

## Supplementary Information

**Additional file 1.**

## Data Availability

The datasets used and/or analysed during the current study are available from the corresponding author on reasonable request.

## References

[1] The Institute for Economics & Peace (IEP) (2018). Global peace index 2018.

[2] (UNHCR) TURA (2018). UNHCR - Syria emergenc.

[3] (ICRC) IC of the RC (2021). Syria crisis: 10 years | ICR.

[4] (SOHR) TSOFHR (2020). Syrian Revolution NINE years on: 586,100 persons killed and millions of Syrians displaced and injured.

[5] Black I (2016). Report on Syria conflict finds 11.5% of population killed or injured, Syria, The Guardian.

[6] Guha-Sapir D, Rodriguez-Llanes JM, Hicks MH, Donneau AF, Coutts A, Lillywhite L, et al. Civilian deaths from weapons used in the Syrian conflict. BMJ (Online). BMJ Publishing Group; 2015;351:h4736. 10.1136/bmj.h4736. 10.1136/bmj.h4736 26419494

[7] Mellor A, Ralph JK, Harrisson SE (2009). Neurosurgical trauma on the battlefield; what can we do and what should we do?. J R Nav Med Serv.

[8] Hodgetts T, Davies S, Midwinter M, Russell R, Smith J, Clasper J (2007). Operational mortality of UK service personnel in Iraq and Afghanistan: a one year analysis 2006–7. J R Army Med Corps.

[9] Agarwalla PK, Dunn GP, Laws ER (2010). An historical context of modern principles in the management of intracranial injury from projectiles. Neurosurg Focus.

[10] Wild H, Barclay •, Stewart T, Leboa C, Christopher •, Stave D, et al. Epidemiology of Injuries Sustained by Civilians and Local Combatants in Contemporary Armed Conflict: An Appeal for a Shared Trauma Registry Among Humanitarian Actors. World J Surg. 2020;44:1863–73. 10.1007/s00268-020-05428-y PMC7223167 32100067

[11] Guha-Sapir D, Schlüter B, Rodriguez-Llanes JM, Lillywhite L, Hicks MHR (2018). Patterns of civilian and child deaths due to war-related violence in Syria: a comparative analysis from the Violation Documentation Center dataset, 2011–16. Lancet Glob Health.

[12] Fouad FM, Sparrow A, Tarakji A, Alameddine M, El-Jardali F, Coutts AP (2017). Health workers and the weaponisation of health care in Syria: a preliminary inquiry for The Lancet-American University of Beirut Commission on Syria. Lancet.

[13] Boseley S (2017). yria “the most dangerous place on earth for healthcare providers” | Syria | The Guardian.

[14] Physicians for Human Rights (2016). Physicians for Human Rights - Syrias Neighbors Must Let Doctors Practice.

[15] World Health Organization (2015). HeRAMS Annual Report January - December 2015 Public Hospitals in the Syrian Arab Republic. Dec 2015.

[16] World Health Organization (2013). Availability of the Health Resources and Services at Public Hospitals in Syria, using HeRAMS.

[17] Taleb Z Ben, Bahelah R, Fouad FM, Coutts A, Wilcox M, Maziak W. Syria: health in a country undergoing tragic transition. Int J Public Health. Birkhauser Verlag AG; 2014;60:63–72. 10.1007/s00038-014-0586-2 25023995

[18] (WHO) WHO (2014). Annual Report 2013 - Syrian Arab Republic | ReliefWeb, OCHA services.

[19] Naaman O, Yulevich A, Sweed Y (2020). Syria civil war pediatric casualties treated at a single medical center. J Pediatr Surg.

[20] Can Ç, Bolatkale M, Sarıhan A, Savran Y, Acara A, Bulut M (2017). The effect of brain tomography findings on mortality in sniper shot head injuries. J R Army Med Corps.

[21] Barhoum M, Tobias S, Elron M, Sharon A, Heija T, Soustiel JF (2015). Syria civil war: Outcomes of humanitarian neurosurgical care provided to Syrian wounded refugees in Israel. Brain Inj.

[22] Jamous MA (2019). Outcome of Craniocerebral Penetrating Injuries: Experience from the Syrian War. J Neurol Surg Part A Cent Eur Neurosurg.

[23] Aras M, Altaş M, Yilmaz A, Serarslan Y, Yilmaz N, Yengil E (2014). Being a neighbor to Syria: A retrospective analysis of patients brought to our clinic for cranial gunshot wounds in the Syrian civil war. Clin Neurol Neurosurg.

[24] McIntyre J (2020). Syrian Civil War: A systematic review of trauma casualty epidemiology. BMJ Mil Health..

[25] Qasaimeh GR, Shotar AM, Alkhail SJA, Qasaimeh MG (2017). The pattern of the Syrian refugee’s injuries managed in King Abdullah University Hospital (Jordan). Eur J Trauma Emerg Surg.

[26] Çelikel A, Karaarslan B, Demirkiran DS, Zeren C, Arslan MM (2014). Suriye’deki savaş esnasında meydana gelen sivil ölümler. Ulus Travma ve Acil Cerrahi Derg.

[27] Teasdale G, Jennett B (1974). Assessment of coma and impaired consciousness a practical scale. Lancet.

[28] Stein SC, Spettell C (1995). The head injury severity scale (HISS): A practical classification of closed-head injury. Brain Inj.

[29] Champion HR, Sacco WJ, Copes WS, Gann DS, Gennarelli TA, Flanagan ME (1989). A revision of the trauma score. J Trauma Inj Infect Crit Care.

[30] Gennarelli T, Woodzin E, Eds. Association for the Advancement of Automotive Medicine. (2016). Abbreviated Injury Scale (c) 2005 Update 2008. Chicago, Illinois; 2016.

[31] Baker SP, O’Neill B, Haddon W, Long WB (1974). The injury severity score: a method for describing patients with multiple injuries and evaluating emergency care. J Trauma.

[32] Boyd CR, Tolson MA, Copes WS (1987). Evaluating trauma care: The TRISS method. J Trauma Inj Infect Crit Care.

[33] Alsaid B, Alhimyar M, Alnweilaty A, Alhasan E, Shalhoum ZAA, Bathich M, et al. Laparotomy Due to War-Related Penetrating Abdominal Trauma in Civilians: Experience from Syria 2011–2017. Disaster Med Public Health Prep. 2020;15(5):615-23. 10.1017/dmp.2020.77 32489173

[34] Action on Armed Violence (2019). Gender and mental health in the Syrian conflict - Syrian Arab Republic | ReliefWeb.

[35] Arafat S, Alsabek MB, Ahmad M, Hamo I, Munder E (2017). Penetrating abdominal injuries during the Syrian war: Patterns and factors affecting mortality rates. Injury.

[36] Hamzeh A, Ayoub R, Issa S, Alhalabi N, Sawaf B, Mohsen F (2021). War-related ocular injuries in Damascus during the Syrian Crisis. Injury.

[37] Hanafi I, Alsalkini M, Husein S, Salamoon M. The delay of breast cancer diagnosis and management during the Syrian war. Cancer Epidemiol. 2023;82:102290. 10.1016/j.canep.2022.102290 36384074

[38] Mateen FJ, Hanafi I, Birbeck GL, Saadi A, Schmutzhard E, Wilmshurst JM, et al. Neurologic Care of Forcibly Displaced Persons: Emerging Issues in Neurology. Neurology. 2023. 10.1212/WNL.0000000000206857. 10.1212/WNL.0000000000206857 PMC10186241 36859408

[39] Karakuş A, Yengil E, Akkücük S, Cevik C, Zeren C, Uruc V (2013). The reflection of the Syrian civil war on the emergency department and assessment of hospital costs. Ulus Travma ve Acil Cerrahi Derg.

[40] Zeren C, Arslan M, Aydogan A, Ozkalipci O, Karakuş A (2012). Firearm injuries documented among Syrian refugees in AntakyaTurkey. Br J Arts Soc Scie.

[41] Adams JH, Graham DI, Murray LS, Scott G (1982). Diffuse axonal injury due to nonmissile head injury in humans: An analysis of 45 cases. Ann Neurol.

[42] Sahuquillo-Barris J, Lamarca-Ciuro J, Vilalta-Castan J, Rubio-Garcia E, Rodriquez-Pazos M (1988). Acute subdural hematoma and diffuse axonal injury after severe head trauma. J Neurosurg.

[43] Gennarelli TA, Thibault LE (1982). Biomechanics of Acute Subdural Hematoma. The Journal of Trauma: Injury, Infection, and Critical Care.

[44] American College of Surgeons the committee on trauma (2018). Student Course Manual ATLS, Advanced Trauma Life Support.

[45] Skaf GS, Elias E. Management of central nervous system war injuries. In: Reconstructing the War Injured Patient. Cham: Springer International Publishing; 2017. p. 131–40.

[46] (UOSSM) U of MC and RO. Syrian Healthcare Research - UOSSM International.[cited 2021 Mar 17]. 2016. Available from: https://www.uossm.org/stats?page=3

[47] Sahloul MZ, Monla-Hassan J, Sankari A, Kherallah M, Atassi B, Badr S (2016). War is the Enemy of Health. Pulmonary, Critical Care, and Sleep Medicine in War-Torn Syria. Ann Am Thorac Soc..

[48] Yun HC, Blyth DM, Murray CK (2017). Infectious complications after battlefield injuries: epidemiology, prevention, and treatment. Curr Trauma Rep Springer Int Publishing.

[49] Jakovljevic M, Al ahdab S, Jurisevic M, Mouselli S (2018). Antibiotic resistance in Syria: a local problem turns into a global threat. Front Public Health.

[50] The World Medical Association Inc (2013). World Medical Association Declaration of Helsinki: Ethical Principles for Medical Research Involving Human Subjects. JAMA.

