# Supplementary material for: War-related traumatic brain injuries during the Syrian armed conflict in Damascus 2014–2017: a cohort study and a literature review
Source: BMC Emerg Med. 2023 Mar 29;23:35. doi: 10.1186/s12873-023-00799-6 (PMC10053936; doi:10.1186/s12873-023-00799-6)

Figures

Supplementary Figure 1. Number of patients recruited in the period between Dec 2014 and Nov 2017.


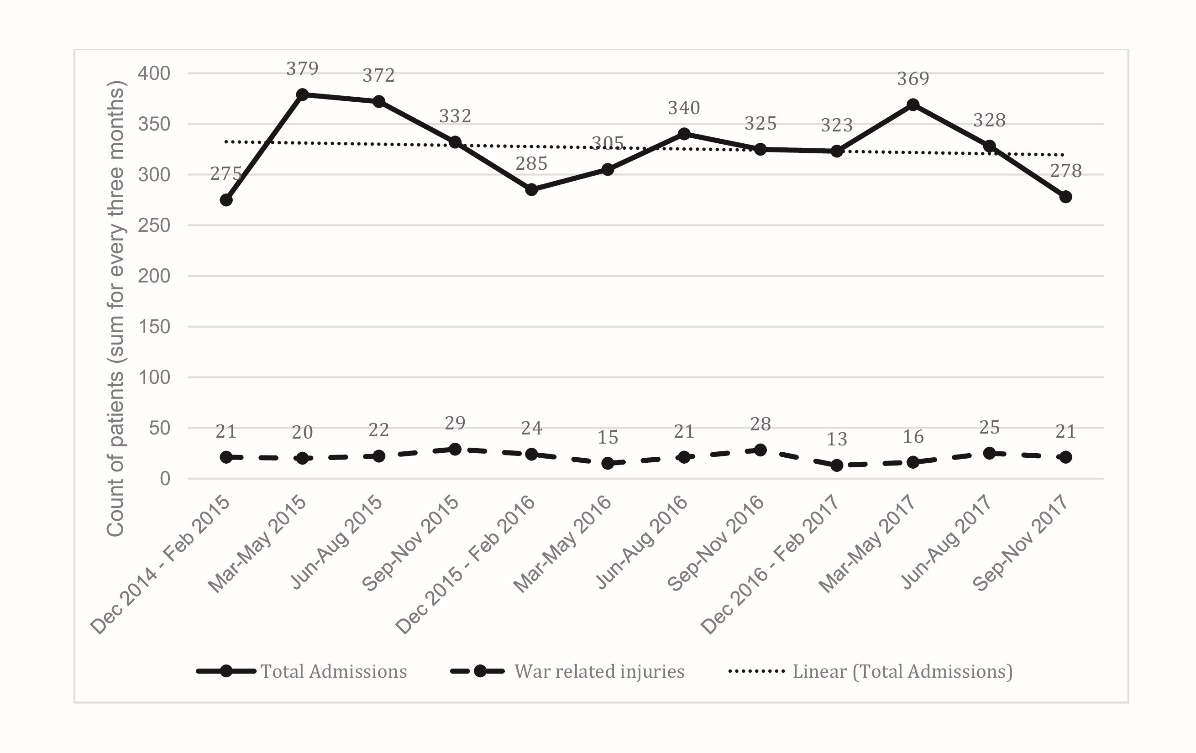


Supplementary Figure 2. Number of victims with war-related traumatic brain injuries.


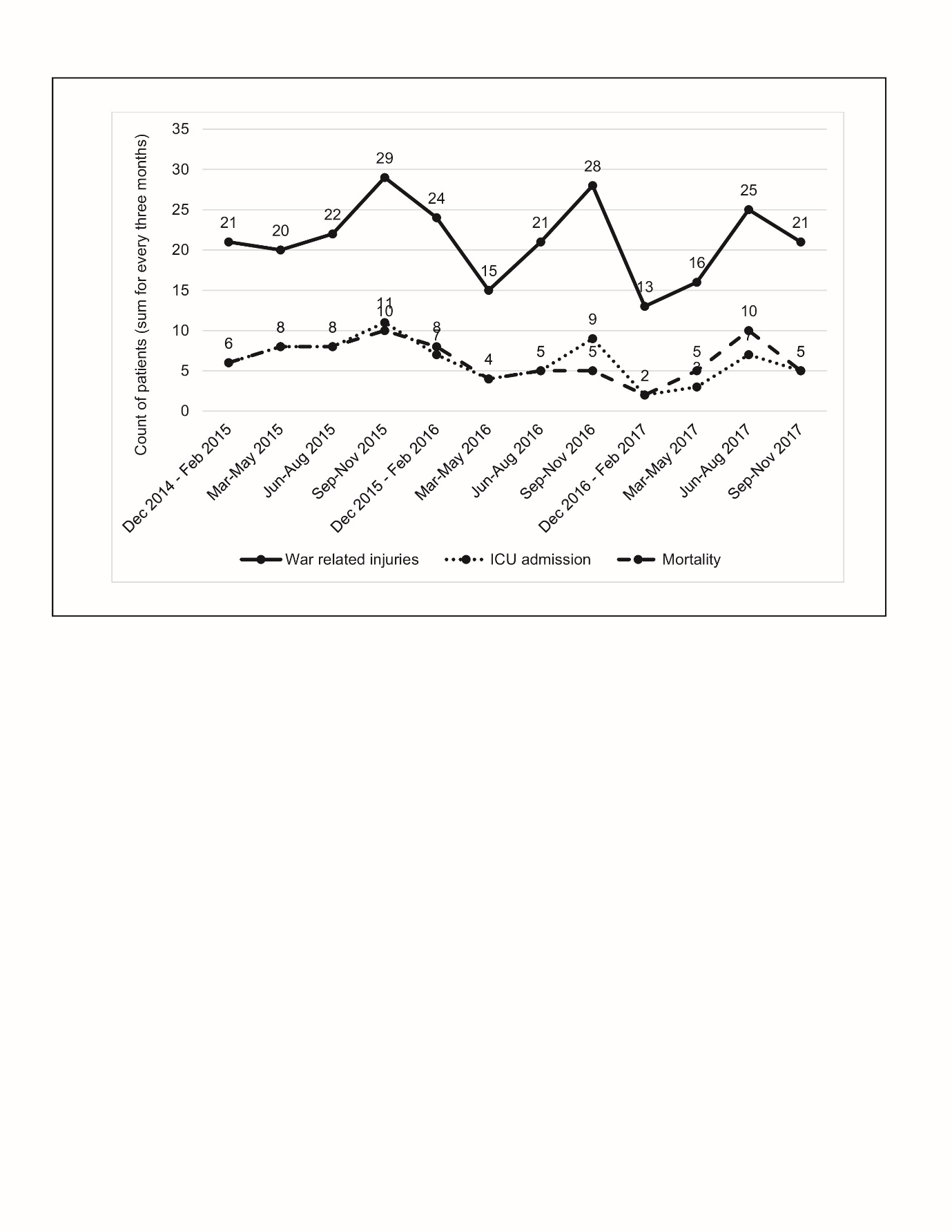

Supplement: Supplementary file 1 — Additional file 1. [file 12873_2023_799_MOESM1_ESM.docx]
